# Bilateral Chronic Cavitary Pulmonary Aspergillosis in a Patient With Mycobacteria Other Than Tuberculosis (MOTT) Infection: A Case Report

**DOI:** 10.7759/cureus.96350

**Published:** 2025-11-07

**Authors:** Ibrahim A Mohamed, Mohamed A Baghi, Vamanjore A Naushad, Basma Ayari, Baraa Mohamed, Qais Al-Said, Ijaz Kamal

**Affiliations:** 1 Internal Medicine, Hamad General Hospital, Doha, QAT; 2 Clinical Department, Weill Cornell Medicine - Qatar (WCM-Q), Doha, QAT; 3 Clinical Department, Qatar University, Doha, QAT; 4 Clinical Department, Q U Health/ Qatar University, Doha, QAT; 5 Clinical Medicine, Weill Cornell Medicine, Doha, QAT; 6 Internal Medicine, Hamad Medical Corporation, Doha, QAT

**Keywords:** aspergilloma, aspergillus flavus, chronic cavitary pulmonary aspergillosis, chronic pulmonary aspergillosis, mycobacterium kansasii, pulmonary aspergilloma

## Abstract

Chronic cavitary pulmonary aspergillosis (CCPA) is a rare, progressive lung infection primarily caused by the* Aspergillus* species. It typically affects individuals with underlying lung conditions, such as pulmonary tuberculosis (TB), cystic fibrosis, chronic obstructive pulmonary disease (COPD), or interstitial lung disease. *Aspergillus fumigatus* is the most commonly identified organism. Clinical manifestations can vary from no symptoms at all to chronic cough with or without hemoptysis. Here, we report a 40-year-old male with bilateral CCPA following nontuberculous mycobacterial (NTM) infection, diagnosed after a persistent history of cough, breathlessness, and episodes of hemoptysis.

## Introduction

Chronic pulmonary aspergillosis (CPA) is an uncommon parenchymal lung disease that often complicates other respiratory conditions [1]. Chronic cavitary pulmonary aspergillosis (CCPA) is the most prevalent form of CPA and tends to be contracted by inhaling the mycotic spores of the *Aspergillus* species and the formation of multiple cavitary lung lesions [1,2]. The diagnosis can be challenging due to the insidious onset and nonspecific symptoms ranging from fever, weight loss, chronic cough to hemoptysis, and progressive shortness of breath [3].

Co-infection with *Mycobacterium* other than tuberculosis (MOTT) further complicates the diagnosis and management of CCPA, as both infections may have overlapping clinical and radiological features. Here, we report a 40-year-old male who presented with poorly controlled type 2 diabetes mellitus, presenting with persistent cough, breathlessness, and hemoptysis. Radiological imaging, including a high-resolution CT scan, revealed bilateral cavitary lesions raising suspicion of fungal etiology. Microbiological evaluation confirmed the presence of *Aspergillus fumigatus* and *Mycobacterium kansasii* from sputum cultures. Further serological tests showed elevated *Aspergillus* antigen levels, supporting the diagnosis. The patient was started on a combination therapy of oral itraconazole, moxifloxacin, azithromycin, and ethambutol, which resulted in gradual clinical improvement and resolution of the symptoms.

## Case presentation

A 40-year-old Indian male presented to the emergency department with a one-day history of hemoptysis associated with a progressively worsening cough, producing purulent greenish sputum for a one-month duration. He additionally reported generalized fatigability over the past few weeks and mild, persistent shortness of breath. He denied any fever, headache, night sweats, skin rash, weight loss, chest pain, joint pain, or hoarseness of voice. His past medical history is notable for diabetes mellitus managed with oral hypoglycemic agents. His family history is unremarkable for pulmonary diseases, malignancy, or connective tissue diseases. He was an active smoker, having quit eight years ago, and had not consumed alcohol. The patient traveled to his home country three months before the onset of his symptoms, but denied any history of contact with sick individuals. He is a driver by profession.

**Figure 1:**
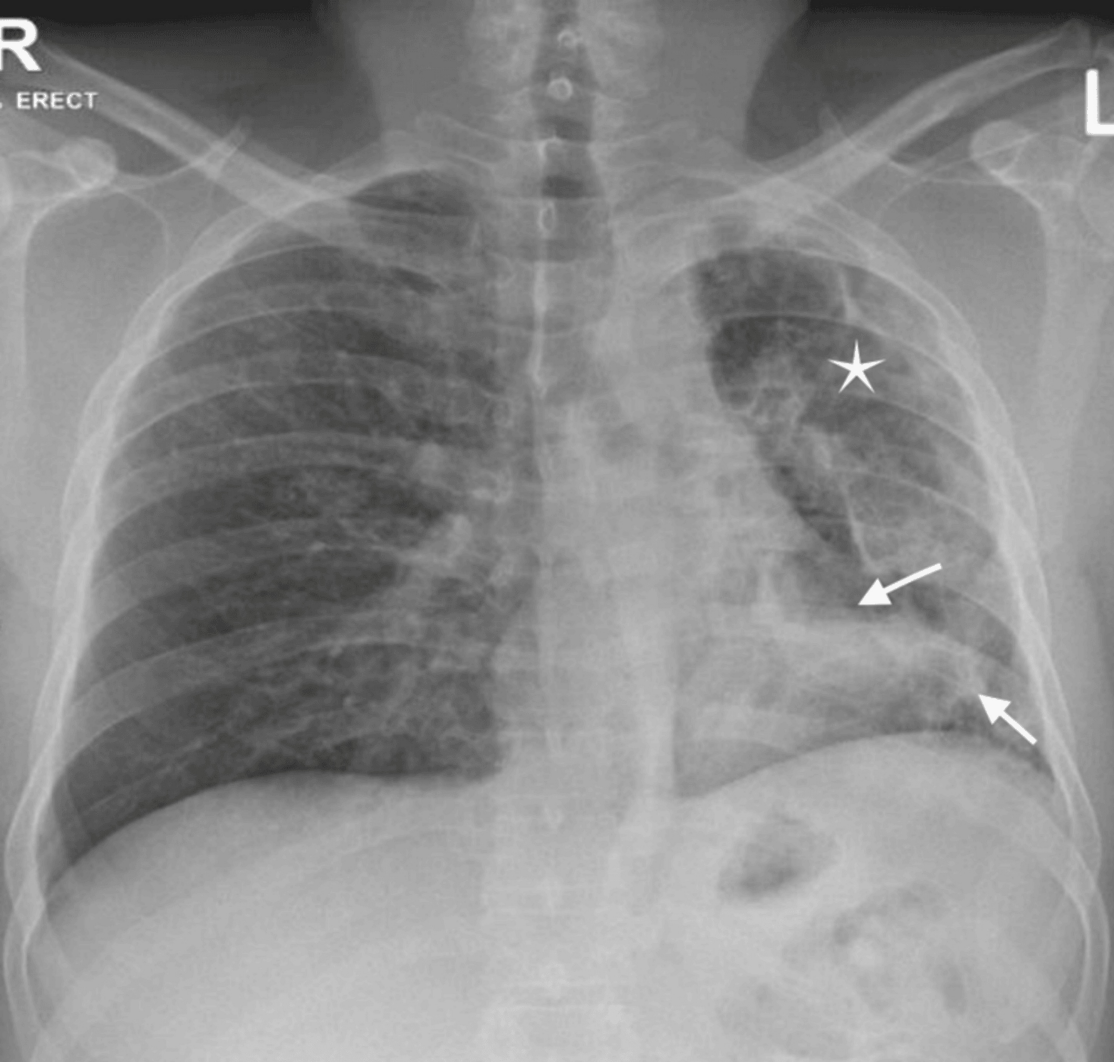
Chest X-ray of the patient Chest X-ray showing reduced left lung volume (white star) and cavitary lesions on the left (white arrows).

**Figure 2:**
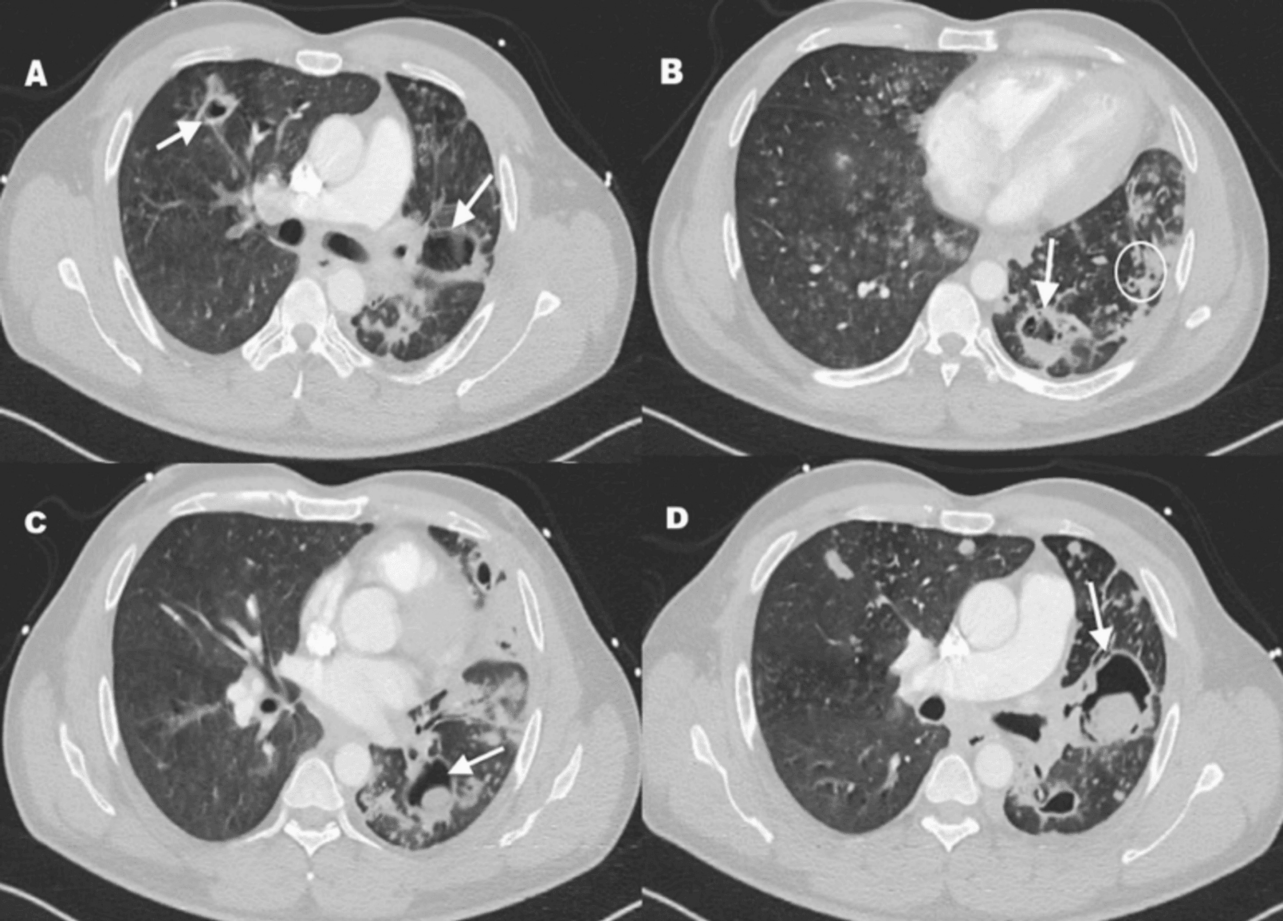
CT image of the patient's chest. Chest computed tomography (CT): Axial views of the lung window showing (a) bilateral cavitary lesions (white arrows), (b) a cavitary lesion in the left lung lobe with a fluid level (white arrow) and cystic bronchiectasis changes (white circle), (c) cavitary lesions with solid components (white arrow), and (d) a large cavitary lesion with irregular wall containing a fungal ball (aspergilloma) within the cavity.

On admission, his vital signs were as follows: pulse rate, 95/min (regular); blood pressure, 110/71 mmHg; respiratory rate, 18/min; oxygen saturation, 99% on room air; and oral temperature, 37.7°C. Initial physical examination revealed bilateral upper extremity digit clubbing without evidence of splinter hemorrhages. Cardiac examination revealed normal heart sounds with no murmurs or gallops. The respiratory system examination showed decreased breath sounds in the left lower lobe, along with coarse crepitations more pronounced on the left side. Initial laboratory investigations revealed leukocytosis (WBC: 11.6 × 103/μL), mild anemia (hemoglobin: 11.7 g/dL), and an elevated C-reactive protein level (72.8 mg/L). Further laboratory investigations revealed poorly controlled diabetes mellitus, with a random blood sugar of 19.2 mmol/L and HbA1c of 10.2% (Table 1). Chest imaging performed on admission, including a chest X-ray and computed tomography (CT) scan, revealed bilateral reticulonodular infiltrates accompanied by cystic bronchiectasis and cavitary lesions, primarily in the left lower lobe. Several of these lesions contained both fluid and solid components, raising suspicion for fungal and bacterial etiologies (Figures 1, 2). 

**Table 1:** Results of the laboratory investigations during hospitalization ANA CTD: anti-nuclear antibody connective tissue diseases; ANCA: antineutrophil cytoplasmic antibodies; NTM: nontuberculous mycobacteria; TB: tuberculosis; PCR: polymerase chain reaction; OD: optical density; BAL: bronchoalveolar lavage; HIV: human immunodeficiency virus; CRP: C-reactive protein

Investigation category	Laboratory details	Result	Reference range
General hematology	White blood cell count (×10^3^/μL)	11.6	4-10
	Hemoglobin (g/dL)	11.7	13.0-17.0
	Red blood cell count (×10^6^/μL)	4.4	4.5-5.5
	Hematocrit (%)	33	40-50
	Platelet count (×10^3^/μL)	269	150-410
Basic metabolic panel	Alanine aminotransferase (U/L)	39	0-41
	Aspartate aminotransferase (U/L)	56	0-40
	Bilirubin T (μmol/L)	7	0-21
	Alkaline phosphatase (U/L)	112	40-129
	Creatinine (mg/L)	1	0.5-1.5
	Urea (mmol/L)	4.2	2.8-8.1
	Sodium (mmol/L)	135	135-145
	Potassium (mmol/L)	3.9	3.5-5.1
	Chloride (mmol/L)	97	95-108
	Calcium (mmol/L)	2.3	2.12-2.60
	Random blood sugar (mmol/L)	19.2	3.3-5.5
	Hemoglobin A1c	10.2	< 6.4
Autoimmune workup	Complement 3 (gm/L)	1.8	0.90-1.80
	Complement 4 (gm/L)	0.30	0.10-0.40
	ANA CTD	Negative	-
	ANCA	Negative	-
Virology	Respiratory viral panel	Negative	-
Microbiology	TB PCR	Negative	-
	Blood cultures	No growth	-
	Sputum culture	*Aspergillus flavus*,* Mycobacterium kansasii*	-
	Acid-fast bacilli (AFB) smear	Mycobacterium kansasii	-
	TB PCR	Negative	
	*Pneumocystis jiroveci *(PCP)	Negative	-
	*Aspergillus galactomannan* antigen test	Positive	-
	*Aspergillus galactomannan* antigen OD	>5.00	< 0.5
Other Special Investigations	BAL culture	*Mycobacterium kansasii*, *Aspergillus flavus*	- -
	HIV Ag/Ab	Negative	
	IgE total (kunits/L)	1,019	<114
	CRP (mg/L)	72.8	<5

The patient was admitted to a negative-pressure room, and broad-spectrum antibiotics, i.e., piperacillin/tazobactam and azithromycin, were initiated. Microbiological testing revealed a positive acid-fast bacilli smear (AFB), but the PCR test was negative, suggesting the possibility of MOTT. The sputum culture yielded positive results for *Aspergillus flavus* and *Mycobacterium kansasii*, whereas two sets of blood cultures showed no growth. Serological tests indicated elevated Aspergillus antigen levels. The autoimmune diseases work-up was negative (Table 1). The infectious disease team was consulted and recommended a combination therapy of oral itraconazole, moxifloxacin, azithromycin, and ethambutol. Bronchoscopy with bronchoalveolar lavage (BAL) was conducted, and the sample tested positive for AFB stain. BAL culture confirmed the presence of *Mycobacterium kansasii *and *Aspergillus flavus*. During the hospital stay, the patient continued to experience episodes of hemoptysis, with a moderate amount of about 60 ml per 24 hours, but without any signs of hemodynamic instability. Tranexamic acid was administered, resulting in partial symptom improvement. The patient subsequently underwent pulmonary artery angiography, which identified non-leaking arteries, and no embolization was performed. On day eight, the patient showed significant improvement, with hemoptysis subsiding. Baseline liver function tests were measured before discharge. He was continued on oral itraconazole, moxifloxacin, azithromycin, and ethambutol to continue therapy for six months, and antidiabetic medications were escalated. During a follow-up two months later, the patient reported significant improvement in shortness of breath and absence of further episodes of hemoptysis. 

## Discussion

CCPA is a form of pulmonary aspergillosis that arises from the colonization and growth of *Aspergillus* in preexisting cavitary lung diseases, such as TB, sarcoidosis, cavitary lung tumors, pulmonary fibrosis, and bronchiectasis [4]. Pulmonary TB is the most prevalent cause of cavitary lesions, which facilitate the formation of aspergilloma [4,5]. Pulmonary aspergillosis manifests in several clinical forms, including invasive pulmonary aspergillosis, chronic necrotizing aspergillosis, allergic bronchopulmonary aspergillosis, CPPA, and aspergilloma [5]. MOTT and *Aspergillus* lung disease are both opportunistic pathogens. The co-infection is significant and associated with a poor prognosis and is rarely reported in the literature [6,7]. MOTT infection is an unusual and challenging clinical entity that can cause pulmonary infections resembling pulmonary TB, but is typically seen in patients with predisposing factors, such as chronic lung diseases or immunosuppressive conditions [7,8]. The most common MOTT species implicated in pulmonary infections include the* Mycobacterium* *avium* complex (MAC), *Mycobacterium intracellulare*, and *Mycobacterium abscessus* [9]. In our patient, both bronchoalveolar lavage and sputum cultures showed the presence of *Mycobacterium kansasii *and *Aspergillus flavus*.* Aspergillus* lung disease typically presents with symptoms, such as persistent cough, hemoptysis, and weight loss, and is often accompanied by cavitary lung lesions visible on radiological studies [8]. Hemoptysis is a severe complication of pulmonary aspergillosis, occurring in 64-83% of cases involving aspergillomas [5]. Bronchial artery embolization can help control significant bleeding and is generally recommended to stabilize patients with severe hemorrhage, especially if medical measures fail to control the bleeding [5]. Surgical resection is considered the most effective treatment for aspergillomas and is typically recommended for patients with a unilateral, localized disease and those who have failed medical treatments [5,6]. In our case, the hemoptysis episodes were managed successfully with tranexamic acid therapy alone, since the patient had uncontrolled DM, which might have been a contributory factor. The diagnosis of pulmonary aspergillosis with concomitant MOTT infection requires a high level of clinical suspicion and exclusion of other possible differential diagnoses, as both conditions can present with similar symptoms, which may potentially delay diagnosis [7]. Moreover, radiographic findings, such as bilateral cavitary lesions, may be observed in both conditions and are not specific to either Aspergillus or MOTT infections, as they can mimic other lung pathologies, including lung neoplasms [7,8]. Microbiological tests, such as sputum and bronchoalveolar lavage cultures, as well as *Aspergillus* serology, play a crucial role in diagnosis.

The treatment of co-infected pulmonary aspergillosis and MOTT infections presents significant therapeutic challenges due to the complexities of both conditions [8]. The major challenge is the potential for drug-drug interactions between antifungal and antimycobacterial medications, adverse effects, and the emergence of drug-resistant organisms [8,9].

The antifungal therapy for pulmonary aspergillosis includes itraconazole or voriconazole [9,10]. Nevertheless, with more extensive infection or failure of oral treatment, intravenous voriconazole or a combination of antifungal agents may be considered [9]. The management of MOTT infections can be complex due to the variety of species and the development of resistance, requiring a combination of antibiotics over extended periods, as untreated infections typically lead to chronic, progressive lung disease [9]. The prognosis for co-infection of CCPA and MOTT, in general, largely depends on the extent of lung parenchymal damage, the underlying immunological status, and the response to treatment [9,10]. In the presented case, a combination of oral itraconazole, moxifloxacin, azithromycin, and ethambutol was used, with a plan to continue the regimen for at least six months, potentially extending to 12 months. The patient's overall response and clinical symptoms showed significant improvement after three months of therapy.

## Conclusions

Co-infection of bilateral CCPA with nontuberculous mycobacteria (MOTT) is a rare and complex clinical condition. Both infections can resemble a variety of pulmonary illnesses because of overlapping symptoms, which can raise suspicion of malignancy and make diagnosis difficult. Our case highlights the importance of considering MOTT co-infection as a possible diagnosis in such challenging cases, especially in patients with comorbidities like diabetes mellitus.

Timely diagnosis and early initiation of suitable antimicrobial and antifungal medications can significantly improve outcomes and avoid complications, as seen in our patient who showed significant progress after combination therapy. A multidisciplinary team, including infectious disease experts, pulmonologists, and radiologists, can further improve prompt diagnosis and reduce misdiagnosis.
